# Peripheral and selective venous liquid biopsy profiling in localized prostate cancer: A pilot feasibility study

**DOI:** 10.1016/j.jlb.2026.100499

**Published:** 2026-10-01

**Authors:** Riccardo Ricotta, Giuseppe Trifirò, Elena Maria Andreolli, Elena Giulia Spinapolice, Elena Tagliabue, Tommaso Ciro Camerota

**Affiliations:** a Oncology Unit, Ospedale Città di Sesto San Giovanni, ASST Nord Milano, Italy; b Nuclear Medicine Unit, Istituti Clinici Scientifici Maugeri IRCCS, Pavia, Italy; c IRCCS MultiMedica, Milan, Italy; d Urology Unit, Casa di Cura San Camillo, Milan, Italy

**Keywords:** Liquid biopsy, Prostate cancer, Selective venous sampling

## Abstract

**Background:**

Liquid biopsy profiling via next-generation sequencing (NGS) is increasingly being used in prostate cancer to identify actionable genomic alterations, monitor disease dynamics, and provide prognostic information. However, the circulating tumor DNA (ctDNA) fraction correlates with disease burden, often remaining near the analytical limit of detection in localized disease. Accordingly, strategies to enhance the molecular characterization in earlier-stage disease represent a clinically relevant priority.

**Methods:**

We conducted a retrospective, single-center observational analysis in a cohort of patients with localized prostate cancer undergoing liquid biopsy. Patients underwent peripheral venous sampling for genomic profiling. When peripheral testing failed to identify genomic alterations or was non-informative, patients proceeded to selective venous blood sampling within the cavo-iliac venous compartment. The primary outcome was the detection rate of reportable genomic alterations in peripheral blood, while exploratory outcomes included findings from selective sampling.

**Results:**

Thirty-one patients were enrolled. Peripheral blood liquid biopsy was evaluable in 28 patients. Genomic alterations were detected in 20 of them (20/28, 71.4%; 95% CI, 51.3–86.8%). Patients most frequently harbored mutations in *ATM* (21.4%), *RB1* (14.3%), and *BRCA1 or BRCA2* (7.1% each). Eight patients underwent selective venous sampling, with 4 out of 8 (50.0%) demonstrating the presence of at least one genomic alteration. Conversion to mutation-positive occurred in two patients (50.0%) who initially lacked detectable alterations in peripheral blood sampling. Across all available peripheral and selective samples, at least one reportable genomic alteration was identified in 23 of the 31 enrolled patients (74.2%).

**Conclusions:**

Our findings demonstrate a high prevalence of genomic mutations in localized prostate cancer. Selective venous sampling was feasible and safe in this small exploratory cohort and provided additional molecular findings in selected patients. These hypothesis-generating data support selective sampling as a potential complementary approach for molecular profiling in localized prostate cancer, deserving prospective validation.

## 1 Introduction

Prostate cancer remains a significant global health burden and a leading cause of cancer-related mortality [1]. In localized disease, risk stratification and treatment selection predominantly rely on conventional clinico-pathological variables, such as serum prostate-specific antigen (PSA) kinetics, multiparametric imaging, and tissue histology [2,3]. The intrinsic genomic heterogeneity present at diagnosis may limit the predictive accuracy of standard clinical models, underscoring the necessity of integrating molecular biomarkers into routine decision-making to achieve a more personalized management approach [4,5]. Circulating tumor DNA (ctDNA), a subset of cell-free DNA (cfDNA), provides a minimally invasive platform for tumor genotyping, longitudinal monitoring, and the assessment of minimal residual disease (MRD) [[6], [7], [8], [9]]. Liquid biopsy has thus emerged as a pivotal tool for analyzing the evolving genomic landscape of prostate cancer [[10], [11], [12], b, [13], a]. Detection is typically performed using next-generation sequencing (NGS), allowing highly sensitive identification of alterations. Liquid biopsy is now established in clinical practice for metastatic castration-resistant prostate cancer (mCRPC), particularly for identifying actionable genomic alterations and monitoring molecular mechanisms of resistance. In contrast, its role in localized disease remains challenging [[14], [15], [16]]. In this setting, the tumor-derived DNA fraction in peripheral blood often falls below the analytical limit of detection due to low shedding and the "dilution effect" of systemic circulation, leading to a high frequency of non-informative or false-negative assays. Although a non-reportable peripheral ctDNA result may reflect a true absence of shedding, it more frequently stems from insufficient ctDNA concentration or inherent analytical constraints [[17], [18], [19]]. Consequently, there is an urgent need for strategies that optimize diagnostic yield and ensure the recovery of high-quality tumor-derived nucleic acids. Recent evidences suggests that venous blood sampled from compartments proximal to tumor drainage may harbor higher ctDNA concentrations before systemic dilution occurs [[20], [21], [22]]. This concept of compartmental enrichment has been explored in other solid malignancies, where tumor-proximal sampling (e.g., portal venous blood) has shown increased ctDNA yield compared to peripheral sites. In the present study, we evaluate the detection rate of genomic alterations in a cohort of patients with localized prostate cancer using the Guardant360 CDx NGS platform [23]. We also describe a novel escalation sampling strategy whereby patients with negative or non-informative peripheral blood results underwent further molecular analysis using samples collected from the central venous compartment through image-guided endovascular selective sampling.

## 2 Materials and methods

### 2.1 Study design and patient population

This study was designed as a single-center, retrospective, observational non-interventional trial. The protocol was approved by the Territorial Ethics Committee Lombardy 6 (Pavia, Italy), and all participants provided written informed consent prior to enrollment. The study reviewed patients assisted between July 2023 and January 2025. Eligible patients included those with histologically confirmed non-metastatic prostate cancer. Staging was performed according to contemporary guidelines using thoracic and abdominal Computed Tomography (CT) and bone scintigraphy, or PSMA PET/CT. All liquid biopsy–based molecular analyses were performed before the initiation of any local or systemic anticancer treatment.

### 2.2 Peripheral blood collection and NGS analysis

Peripheral whole blood (10 mL) was collected via venipuncture into streck cell-free DNA blood collection tubes (BCTs) to stabilize extracellular DNA and minimize contamination from genomic DNA released by leukocyte lysis. Circulating cfDNA was analyzed using the Guardant360 CDx (Guardant Health, Inc., Redwood City, CA) assay, a semi-automated, NGS-based in vitro diagnostic platform. The assay utilizes targeted high-throughput hybridization-based capture technology to detect single nucleotide variants (SNVs), insertions, and deletions (indels) in 74 genes. Copy number amplifications (CNAs) in 18 genes, fusions in 6 genes, microsatellite instability (MSI) status.The presence of mutations was quantified as the variant allele frequency (VAF), defined as the percentage of cfDNA fragments harboring a specific alteration relative to the total cfDNA at that genomic position. Samples were processed in the central laboratory within 7 days of collection.

### 2.3 Selective venous sampling procedure and NGS analysis

Patients with negative or non-informative peripheral liquid biopsy results, and no clinical contraindications, were considered for image-guided selective venous sampling. Peripheral results were classified as negative when no reportable genomic alterations were detected and as non-informative when pre-specified assay quality-control criteria were not met. Selective venous sampling was therefore used as an exploratory, pragmatic escalation strategy in these cases, with the aim of reducing the impact of potential pre-analytical failures and increasing the likelihood of recovering adequate tumor-derived material for molecular analysis. In our study, liquid biopsy was performed via selective percutaneous catheterization of veins draining the effluent circulation of a neoplasm by interventional radiologists or vascular imaging specialists. The procedure can be carried out in an outpatient setting under local anesthesia, it is repeatable without significant limitations and is generally not associated with major complications. Femoral vein access is favored due to the vessel's caliber, superficial location, and anatomical landmarks, particularly its consistent proximity to the femoral artery. As a result, ultrasound guidance is not always mandatory. Selective venous blood samples for liquid biopsy were obtained bilaterally from the internal iliac veins. In three cases, for anatomical reasons, the orifice of internal iliac vein has been not reached and blood sample has been obtained in the proximity of the supposed position of the internal iliac vein. The catheters used are those routinely employed in diagnostic or interventional angiographic procedures and selective cannulation of tumor-draining veins, even in multiple sites, can be achieved under fluoroscopic guidance. 10 mL tubes containing a ctDNA stabilizing agent are collected through a single catheterization via one of the femoral veins. Samples are stored at room temperature and must be delivered to the laboratory within 7 days for NGS analysis.The catheters typically used are Cobra-shaped (C1 or C2), selected according to the patient's vascular anatomy. The reproducibility of bilateral catheterization using the cross-over technique approaches 100%, with a total procedure time of approximately 15 min and an average radiation exposure of less than 2 mSv.

### 2.4 Statistical analysis

Descriptive analyses were performed. Continuous variables were reported as medians and interquartile ranges and stratified by blood sampling site, namely peripheral and selective venous sampling. The percentage of patients harboring any genomic alteration was also calculated. Box plots were used to visualize overall VAF distributions in the two groups. No formal statistical comparisons were performed because of the small number of observations.

### 2.5 Study objectives

The primary objective of this study was to determine the detection rate of genomic alterations in peripheral blood using the Guardant360 CDx NGS assay in a cohort of patients with localized prostate cancer.

Exploratory endpoints included:

- Conversion rate: The proportion of patients with initially negative or non-informative peripheral results who subsequently demonstrated detectable alterations upon selective venous sampling
- Cumulative molecular detection yield: the percentage of patients with detectable alterations identified through the implementation of selective venous sampling
- Feasibility and diagnostic relevance of selective sampling

## 3 Results

A total of 31 subjects were included. Patients characteristics are summarized in Table 1. Peripheral sample testing was performed in 29/31 patients. One sample was non-informative. Overall, 20/28 patients harbored at least one detectable genomic alterations, yielding an overall positivity rate of 71.4% (95% CI, 51.3–86.8%). Up to three distinct gene alterations per subject were identified. Four patients harbored alterations in three different genes, six patients in two genes, and the remaining ten patients in a single gene. ATM serine/threonine kinase (*ATM*) was the most frequently altered gene (6 patients, approximately 21.4%), followed by RB transcriptional corepressor 1 (*RB1*) (4 patients, approximately 14.3%). Mutations involving APC regulator of WNT signaling pathway (*APC*), BRCA1 DNA repair associated (*BRCA1*), BRCA2 DNA repair associated (*BRCA 2*), discoidin domain receptor tyrosine kinase 2 (*DDR2*), and tumor protein p53 (*TP53*) were each detected in 2 subjects (approximately 7.1%). The genomic landscape found by peripheral blood sampling was shown in Table 2. A total of eight patients underwent selective venous sampling, yielding 17 blood samples overall (one to four per patient), of which 14 (82.4%) were obtained from the internal iliac veins. Among them, genomic alterations were detected in four patients (50.0%). More specifically, two of these patients had previously undergone peripheral blood testing that yielded no detectable mutations, while one patient had not undergone prior peripheral blood analysis. In the remaining patient, selective sampling confirmed the presence of the same mutation previously identified in peripheral blood. Of note, a conversion rate from negative peripheral testing to positive selective sampling was observed in 2/4 cases (50.0%). Within this cohort AT-rich interaction domain 1A (*ARID1A1*) was the most common mutation, being identified in 2 out of 8 patients (25%).The genomic landscape found by selective blood sampling was shown in Table 3. Overall, molecular alterations were detected in 23 of 31 patients (74.2%), including both patients with detectable alterations in peripheral blood and those in whom alterations were identified only through selective sampling. We reported a patient-level descriptive overview of genomic findings obtained from selective venous sampling together with paired peripheral blood results, when available. Detected genomic alterations, corresponding VAFs, and sampling sites are summarized in Table 4. Below, we describe the frequency and VAF of the genomic alterations detected in peripheral and selective blood samples. Peripheral blood NGS identified 34 genomic alterations across 20 patients, with a median VAF of 0.38% (IQR, 0.19–1.16%). A total of 7 mutations were identified in in the eight selective samples, with median VAF of 0.52% (IQR 0.33–0.61%). (Fig. 1). The assessment of any associations with clinical risk variables (e.g., baseline PSA and ISUP grade) and paired comparisons in patients undergoing both selective and peripheral sampling, were not addressed because of the limited sample size, the small number of matched evaluable cases, and the resulting lack of statistical power. Lastly, in addition to its diagnostic yield, the feasibility and safety of selective blood sampling were specifically assessed. Notably, no procedure-related complications were reported among patients who underwent selective sampling, supporting the favorable safety profile of this procedure.

**Table 1:** Demographic and clinicopathological data.

Characteristic	Value
Patients, n	31
Age, median (IQR), years	71.2 (68.4–74.3)
PSA, median (IQR), ng/mL	9.0 (6–15)
ISUP grade group at biopsy, n (%)	
1	5 (16.1)
2	11 (35.4)
3	9 (29)
4	1 (3.2)
5	5 (16.1)

Abbreviations: IQR = interquartile range; ISUP= International Society of Urological Pathology.

**Table 2:** Distribution of genomic alterations in peripheral blood samples.

Gene	No. of mutations	% of total mutations	Alterations detected
*ATM*	6	17.6	P2353L; c.3077+1G > A; R2832H; R717Q; S2408L; I124V
*RB1*	4	11.7	K475R; S855R; c.265-2A > G; Q62
*APC*	2	5.9	W593S; I1987V
*BRCA1*	2	5.9	S1461N; S840I
*BRCA2*	2	5.9	c.7431_7435 + 14delTTTAGGTATTGTATGACA; L1908L
*DDR2*	2	5.9	D728D; L777L
*TP53*	2	5.9	Y220C; V197L
*ARID1A*	1	2.9	A339A
*CDH1*	1	2.9	R74Q
*CDK12*	1	2.9	I897I
*CDK4*	1	2.9	P256L
*CDKN2A*	1	2.9	V126I
*ERBB2*	1	2.9	V424I
*HNF1A*	1	2.9	T196T
*JAK2*	1	2.9	V617F
*KIT*	1	2.9	P754S
*MAPK3*	1	2.9	N274S
*MET*	1	2.9	N371N
*MYC*	1	2.9	Amplification
*NRAS*	1	2.9	I36T
*PDGFRA*	1	2.9	E156*

**Table 3:** Distribution of genomic alterations in selective blood samples.

Gene	No. of mutations	% of total mutations	Alterations detected
*ARID1A1*	2	28.6	A339A; S1350F
*BRCA2*	1	14.3	V290_D293
*GATA3*	1	14.3	S403C
*KRAS*	1	14.3	G13D
*NTRK1*	1	14.3	A337A
*RB1*	1	14.3	K475R

**Table 4:** Patient-level genomic findings from selective venous sampling with paired peripheral blood results.

Patient ID	Peripheral blood		Selective venous sampling			Interpretation
	Result	Genomic alteration(s), VAF (%)	Result	Sampling site(s)	Genomic alteration(s), VAF (%)	
1	Reportable alterations detected	*ARID1A* S1350F, 0.49% *RB1* K475R, 0.37% *ATM* P2353L, 0.12%	Reportable alterations detected	Inferior vena cava	*ARID1A* S1350F, 0.61% *RB1* K475R, 0.52%	Partial concordance: *ARID1A* and *RB1* detected in both peripheral and selective samples; *ATM* detected only in peripheral blood
2	No reportable alterations detected	None detected	Reportable alteration detected	Bilateral external iliac veins Bilateral internal iliac veins	*ARID1A* A339A, 3.8% (left external iliac vein) No reportable alterations detected in the remaining selective samples	Reportable alteration detected only on selective sampling
3	No reportable alterations detected	None detected	No reportable alterations detected	Bilateral internal iliac veins	None detected	No reportable alterations detected in either peripheral or selective samples
4	Non-informative	-	No reportable alterations detected	Bilateral internal iliac veins	None detected	Peripheral assay non-informative; no reportable alterations detected on selective sampling
5	Not performed	-	Reportable alterations detected	Bilateral internal iliac veins	*NTRK1* A337A: right 0.58%; left 0.58% *GATA3* S403C: right 0.28%; left 0.33% *BRCA2* V290_D293dup: left 0.48%	Peripheral testing not performed; bilateral detection of *NTRK1* and *GATA3* on selective sampling, with *BRCA2* detected only in the left internal iliac vein sample.
6	Not performed	-	No reportable alterations detected	Bilateral internal iliac veins	None detected	Peripheral testing not performed; no reportable alterations detected on selective sampling
7	No reportable alterations detected	None detected	Reportable alteration detected	Bilateral internal iliac veins	*KRAS* G13D: right 0.33%; No reportable alterations detected in the left internal iliac vein sample.	Reportable alteration detected only on selective sampling
8	No reportable alterations detected	None detected	No reportable alterations detected	Bilateral internal iliac veins	None detected	No reportable alterations detected in either peripheral or selective samples

**Abbreviations:** VAF, variant allele frequency.

**Notes:** Patient 1-8 denote anonymized study participants; Gene symbols are shown in italics; 'None detected' indicates that no reportable genomic alteration was identified in an evaluable sample; 'Non-informative' indicates that the assay did not meet pre-specified quality-control criteria; 'Not performed' indicates that peripheral testing was not undertaken.

**Fig. 1:**
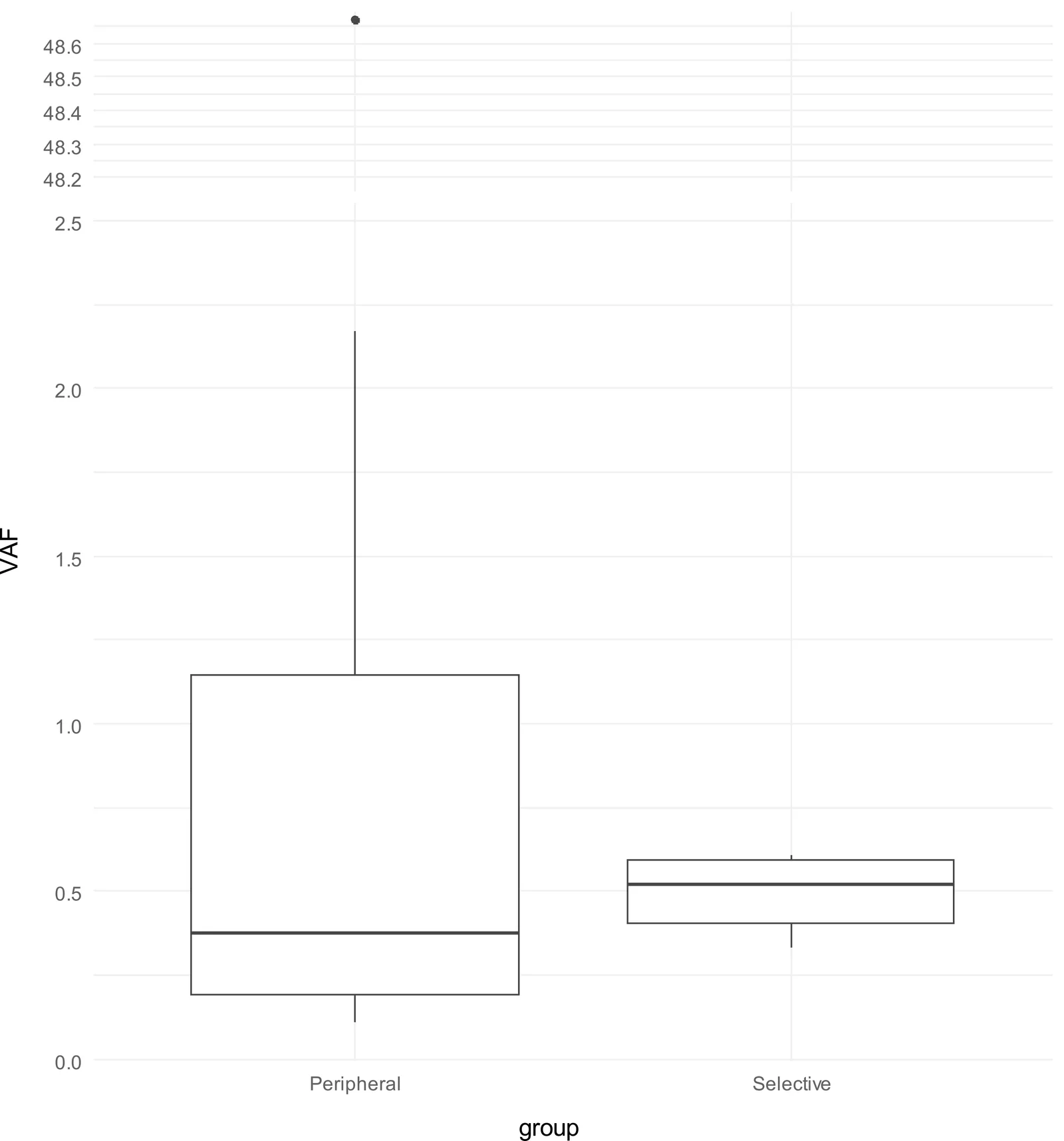
Box plot comparing VAF distribution between peripheral and selective sampling groups.

## 4 Discussion

Recent studies underscore the clinical value of liquid biopsy in advanced prostate cancer, while also highlighting limited detectability as both a biological and analytical constraint [[24], [25], [26], [27]]. In castration-sensitive disease (CSPC) ctDNA abundance tends to track with metastatic burden and clinical outcomes. In this setting, real-world genomic alteration signatures have been linked to progression-free outcomes, reinforcing the prognostic information encoded in plasma-derived genomic profiles when ctDNA is detectable. In metastatic CRPC, ctDNA dynamics are increasingly recognized as markers of disease evolution [[28], [29], [30], [31]]. Changing metastatic patterns have been associated with shifts in ctDNA levels, suggesting that both anatomic distribution and clonal evolution influence the amount and composition of ctDNA released into circulation. Consequently, the absence of detectable mutations on ctDNA analysis does not definitively rule out the presence of tumor-associated molecular abnormalities. The challenge in localized prostate cancer is the limited ability of peripheral plasma assays to capture them reliably at very low tumor fractions. Across cancers, ctDNA detection is strongly stage-dependent, with early-stage disease frequently falling below detection thresholds [16,32]. Nevertheless, liquid biopsy is emerging as a promising non-invasive strategy for diagnosis, risk stratification, minimal residual disease assessment, treatment planning, and identification of actionable molecular alterations. Importantly, this approach may be particularly valuable when histological tissue is unavailable, insufficient, or unsuitable for adequate molecular characterization. In addition, the minimally invasive nature of liquid biopsy makes it suitable for serial assessments, allowing longitudinal evaluation of tumor burden, molecular disease dynamics, clonal evolution, response to treatment, and emergence of resistance alterations over the course of disease. CtDNA abundance in plasma is influenced by tumor burden, proliferative and apoptotic rates, vascularization, and anatomic drainage patterns. Localized prostate tumors and limited nodal disease often release ctDNA at levels near or below the detection thresholds of standard NGS workflows, particularly when broad panels are used and VAFs fall below ∼0.1–0.5%. Current ESMO recommendations underscore that low concentrations of plasma ctDNA may compromise variant detection, emphasizing that a negative result does not definitively exclude the presence of tumor-derived genomic alterations [33]. Expert consensus and international guidelines, advocates for reflex testing following a non-informative liquid biopsy to mitigate the inherent risk of false-negative findings [[34], [35], [36]]. A novel stepwise approach with escalation to selective venous sampling aims to shift the pre-test probability of detection by exploiting anatomic drainage and minimizing avoidable pre-analytical loss. Central venous sampling provides a biologically grounded opportunity for enrichment within the same patient. This approach conceptually parallels tumor-proximal sampling in other malignancies and may be relevant in prostate cancer where venous drainage from pelvic structures may transiently concentrate tumor-derived fragments. Selective venous sampling aims to obtain a higher concentration of ctDNA for molecular analysis, avoiding dilution of the biomarker in the systemic circulation, as occurs with peripheral blood sampling. The analytical advantage of this approach has long been recognized in critical biochemical assays, such as hormonal testing applied to the diagnosis of pituitary tumors, for which inferior petrosal sinus catheterization has historically been performed. Similarly, in adrenal tumors, selective sampling from the adrenal veins is performed, whereas in pancreatic insulinomas, blood is collected from the hepatic veins. These vascular districts can be accessed via percutaneous catheterization through brachial or femoral venous routes. Blood sampling during open surgery, performed directly from the pulmonary veins for lung tumors and from the portal vein for abdominal malignancies, has demonstrated both quantitative and qualitative analytical enrichment, due to reduced degradation and shorter circulation time of biomarkers such as circulating tumor cells, ctDNA, and extracellular vesicles [37]. To date, robust clinical series evaluating sampling of the prostatic/internal iliac venous drainage in patients with localized prostate cancer are lacking. Consistent with findings reported in other solid tumors, we incorporate the selective sampling as an escalation approach in patients with no reportable molecular alterations detected in peripheral blood, with the aim of exploring whether additional molecular information could be recovered in a low-shedding setting. Several mechanistic hypotheses could explain improved yield from a central compartment: (i) proximity to venous drainage from pelvic tumor sites and regional metastases, (ii) reduced dilution by systemic blood volume at the moment of sampling, and (iii) potential enrichment of tumor-derived nucleic acids prior to first-pass clearance or further dilution. While the “tumor-proximal” sampling paradigm has stronger precedent in other malignancies (e.g., portal venous sampling in pancreatic cancer, pulmonary venous sampling in lung cancer), the principle is biologically plausible for pelvic malignancies as well. In prostate cancer, strategies to improve ctDNA detection have also included timing-based approaches, such as increased ctDNA detection in a post-biopsy window. Our approach differs by attempting an anatomic sampling modification rather than a temporal one. Notably, our escalation strategy was deployed only after a negative peripheral result, aligning with an efficiency principle: reserve invasive sampling for those in whom the marginal value is potentially highest. Even in the absence of an immediate indication for targeted therapy, early identification of selected tumor alterations, such as DNA repair gene abnormalities, may still have relevant practical implications for patients. It may help identify individuals harboring genomic features associated with more aggressive disease biology, could enable risk-adapted intensification or de-intensification strategies, prompt hereditary cancer risk assessment, and facilitate early enrollment in biomarker-driven clinical trials [38]. The present study shows that peripheral liquid biopsy is informative in the majority of patients with localized prostate cancer. Recently, Bettoni et al. reported similar findings in a small cohort of patients with high-risk localized prostate cancer, with genomic alterations detected in 89% of plasma samples. Notably, only 54.5% of tissue biopsies yielded useable sequencing data, while tissue–plasma concordance could not be robustly assessed because only four matched pairs were available [39]. Our findings provide a preliminary biological signal that sampling closer to the tumor-draining compartment may enrich the tumor-derived fraction by peripheral blood testing. The integration of peripheral and selective venous sampling resulted in reportable genomic alterations being detected in 74.2% of the overall study cohort. Given the exploratory design and the limited number of patients undergoing selective venous sampling, this finding should be interpreted descriptively and does not establish a diagnostic advantage over peripheral blood testing. Rather, selective venous sampling may represent a complementary approach for obtaining additional molecular information in selected patients, particularly when peripheral liquid biopsy is negative or non-informative. Notably, selective venous sampling was associated with a favorable procedural safety profile, with no procedure-related complications observed across the study cohort. Several limitations should be acknowledged. The small cohort and the limited availability of paired peripheral and selective samples prevented a robust comparative analysis and restricted the assessment of potential associations between genomic alteration detection and clinical variables, including PSA levels and ISUP grade. In addition, the absence of paired tissue sequencing prevented the evaluation of sensitivity and specificity against a reference standard. Accordingly, these findings should be regarded as hypothesis-generating and warrant further validation in larger prospective cohorts. Despite its potential to provide additional molecular information in selected patients, the implementation of selective venous sampling also entails relevant practical considerations. The procedure requires specific interventional radiology expertise, dedicated infrastructure and organizational resources, as well as appropriate patient selection. These requirements may limit its broader scalability and currently make this approach more suitable for selected clinical or research settings than for widespread routine use. The magnitude of molecular enrichment may also vary according to tumor location, nodal involvement, and individual venous anatomy. Finally, the costs associated with NGS analysis may represent an additional barrier to broader implementation. Prospective multicenter studies are therefore needed to quantify the incremental diagnostic yield of this approach and to define minimal procedural requirements that ensure safety, reproducibility, and feasibility in a real-world scenario.

## 5 Conclusions

This study demonstrates that liquid biopsy is highly effective and capable of identifying genomic alterations in a large fraction of patients with localized prostate cancer. In this small exploratory cohort, selective venous sampling was feasible and safe and provided complementary molecular information in selected patients. These preliminary findings should therefore be considered hypothesis-generating and warrant validation in a prospective paired-sampling study specifically designed to assess whether the selective venous sampling should remain a niche rescue procedure or can be integrated into broader precision-oncology pathways in localized prostate cancer.

## Data availability statement

The individual-level clinical and genomic data generated and analyzed during the current study are not publicly available due to patient privacy, ethical, and data protection restrictions. De-identified data supporting the findings of this study may be made available by the corresponding author upon reasonable request, subject to approval by the relevant institutional and ethics authorities and in accordance with applicable data protection regulations.

## Author contributions

Conceptualization: GT,TCC; Data curation: EMA, TCC; Formal analysis: ET; Investigation: TCC, EGS, GT; Visualization: ET, RR; Writing original draft: RR, TCC; Review & editing: EMA, EGS, ET, GT.

## Ethics declaration

Written informed consent to take part in the study and to publish the article has been obtained from all participants or their legal representatives. The privacy rights of participants have been observed.

This study included organ or tissue donors.This study includes human biological material and consent was obtained by donors, or their next of kin or legal representatives, for use in this study and for publication of the article. The samples used in this research were not sourced from executed prisoners or prisoners of conscience.

This study was performed in compliance with relevant laws, regulatory frameworks and guidelines where the research took place.This study was approved by the Territorial Ethics Committee Lombardy 6, Italy.(Approval No. CTSPV52-24)

## Financial support

This study has been financially supported by Fondazione Falciani ETS, Milan, Italy.

## Declaration of competing interest

The authors declare that they have no known competing financial interests or personal relationships that could have appeared to influence the work reported in this paper.
